# Selenium nanoparticles effect on foot and mouth disease vaccine in local Awassi breed male lambs

**DOI:** 10.5455/javar.2024.k785

**Published:** 2024-06-08

**Authors:** Aseel M. Hamzah, Tamara N. Dawood

**Affiliations:** 1 Zoonotic Disease Unit, Veterinary Medicine College, Baghdad University, Baghdad, Iraq; 2 Department of Public Health, College of Veterinary Medicine, Baghdad University, Baghdad, Iraq

**Keywords:** Selenium nanoparticles, FMD vaccine, ELISA

## Abstract

**Objective:**

The goal of this research was to evaluate where selenium nanoparticles impact the activity of antibodies in immunized lambs with foot and mouth vaccines by modulating the immune system.

**Materials and Methods:**

Two groups of lambs of 3–4 months of age were injected with 1 ml of ARRIAH-VAC vaccine intramuscularly in the neck, five Lambs were given selenium nanoparticles (size 100 nm) oral administration of selenium nano dose of 0.1 mg/kg of body mass once every day for sixty days considered as group one (G1) while the other five used as control Group 2 (G2).

**Results:**

This resulted in the establishment of an immune response, as evidenced by a rise in antibody titer in the blood using the ELISA test for three serotypes A, O, and Asia 1, when selenium nanoparticles were given orally at a dose of 0.1 mg/kg body weight after immunization, we noticed a significant (*p* >0:05) selenium nano group increase in IgG response in all immunized groups in contrast to lambs that had only received the foot-and-mouth disease vaccine

**Conclusion:**

We have demonstrated that selenium nanoparticles administered orally significantly enhance immune responses while also increasing body weight.

## Introduction

As a widespread viral animal disease that kills young animals and reduces adult animals’ productivity, foot-and-mouth disease (FMD) continues to be regarded for its negative social and economic effects as well as its financial costs [1]. The first confirmed FMD cases were recorded in 1937, although serotype (A) of FMD disease was not discovered until 1952 in Iraq. Since then, a number of serotypes have been identified; the serotypes O, SAT-1, and Asia 1 were identified in 1957, 1962, and 1975, respectively, and various studies from 1999 found that the most prevalent serotypes found in Iraq between 1952 and 1998 were serotype A, O, Sat1, and Asia 1A/ASIA/G-VII [G-18] is the name of the new serotype A lineage [2–5]. Nonetheless, Iraq suffers from an annual outbreak of FMD that costs livestock owners a lot of money. The disease affects cattle, buffalo, and small ruminants, with the number of sick and dead animals changing, the sickness reappeared in 2011 and affected 13,305 small ruminants, 6,757 cattle, and 5,216 buffalo. The veterinary clinic initiated a vaccination program in 2011, and about 1,935,510 buffalo, cattle, and 7,062,003 small ruminants, in that order, got immunizations, despite the fact that fewer animals were impacted overall. While the number of diseased animals was lower in 2012 when FMD outbreaks returned to fifteen Iraqi governorates, the veterinary service also launched a vaccination campaign that year, immunizing over 7,105,941 and 1,798,074 bovines and ovines, respectively. The number of animals infected with disease was noticeably declining in 2013, 2014, and 2015 while the prevalence rate for bovine and ovine increased in 2016 compared to 2015, according to the veterinary service [6].

The immunomodulatory effects of selenium nanoparticles were assessed in this work, producing long-lasting humoral and cellular immunological responses to boost vaccination-induced immune response [7–9]. Nanoselenium is less toxic as well as more suitable for biological use than sodium selenite, and it also has a number of helpful features, including excellent antioxidant activity, adsorption strength, surface activity, and catalytic performance [10–13]. Antioxidants selenium (Se) provide cell protection via reducing free radicals and preventing lipid peroxidation, from reactive oxygen (RO) [14,15] as well as the activity of glutathione peroxidases depends on selenium (Se) which enzymes are capable of reducing lipid hydroperoxides and hydrogen peroxide [16]. Additionally, Selenium serves an important role in a number of substances that alter the production of specific cytokines or improve immunological cells that are resistant to oxidative damage, both of which are known to enhance immune system response [17,18]. The amount of selenium that an animal needs relies on the minerals that are present in the soil, as well as the qualities of the food that is grown and fed to animals. The reaction to neonatal survival and juvenile animal production efficiency is contingent upon the concentration of selenium inside the animal body [19]. A supplement substitute that can provide benefits that enable needs to be satisfied by constant element release is the use of ruminal bolus containing prolonged-release nanoparticles [20]. Furthermore, Nanoselenium looks to be less toxic and more biocompatible than sodium selenite, in addition to having several beneficial qualities such as high antioxidant activity, adsorption strength, surface activity, and catalytic performance [21,22]; additionally, humoral immunity is also increasing [23,24].

Current research is therefore focused on the application of selenium nanoparticles to target enhancing the local male lambs’ immune response against FMD vaccine. In particular, the experiment was designed to test whether or not selenium nanoparticles alter immunity in FMD antibody-bearing lambs that had just been injected with the vaccine. This indicates that when selenium nanoparticles were given orally following immunization, there was an increase of IgG in all the groups compared to lambs which only received FMD vaccine. This study shows that selenium nanoparticles can be a useful way to boost immune responses in vaccinated lambs.

The objective of the current research was to determine the bioavailability of selenium (Se) in lambs following its administration in the form of Se nanoparticles after vaccinating the lamb with the FMD vaccine. The goal of the current work is to evaluate the new biosynthesis of Se-nanoparticles to increase body weight and potent the immune system.

## Material and Methods

### Ethical statement

College of Veterinary Medicine’s Institutional Animal Care and Use Committee approved every treatment performed on the study’s animals (Reference number: P.G. 1301).

### Bacterial culture

In 250 ml of nutrient broth, a loopful of *P. aeruginosa* was injected, where it was then left to incubate for 24 h at 37°C. Centrifugation was utilized to eliminate bacterial cells at 6,000 rpm for 10 min, after which liquid was gathered and placed in a separate 250-ml container.

### Selenium nanoparticle synthesis

Bacterial cultivation in a 250 ml flask that had been previously prepared as described within the “Bacteria” portion, which includes simply Bacteria and media, was mixed with Na_2_SeO_3_ at a concentration of 2 mM for the nanoparticle formation. The bacteria were incubated for 72 h at 37°C after being injected with the metallic salt and 200 rpm in a shaking incubator. Within three days of the experiment, NB media’s typical yellowish color changed to orange-red, showing that Se had been reduced to elemental selenium then overall samples were centrifugation at 6,000 rpm for fifteen minutes after 72 h after that the particle was washed twice with deionized distal water after the supernatant was removed then the sediment put in glasses petri dish and incubated at 37°C for dryness.

### UV-visible spectra analysis

The generated spectrum of selenium nanoparticles was assessed using a JASCO 670-UV-Vis spectrophotometer with a resolution of 0.86 nm and a wavelength range of 200 to 1,200 nm at ambient temperature [25,26].

### Fourier transform infrared (FTIR)

The produced selenium nanoparticles were examined by FTIR spectroscopy as explained in [27].

### X-ray diffraction (XRD)

XRD (Shemadzu-6000 Japan) was used to characterize the crystalline structure of nanoparticles [28,29].

### The experimental animal

The study was conducted at the Field College of Veterinary Medicine/University of Baghdad. We employed ten healthy males (local Awassi lambs) that were 3 months old and weighed, on average, between 18.5 and 20 kg. In contrast to the first group (G1), taken once a day by mouth at a dosage of 0.1 mg/kg of selenium nanoparticles for eight weeks while group 2 (G2) is considered as the control group. The animals were kept in the field of the veterinary medicine college and were given access to water and a daily feeding of concentrated nutrition that represents two percent and half of each body mass per head (Table 1) with straw and hay.

**Table 1.** The composition of the elements in a concentration diet.

“Nutritional ingredients”	%
Wheat bran	20
Corn	20
Barley	48
Soya bean	10
Premix	1
Salt	1
% Total	100

### Blood samples

From the beginning until the end of the project, as well as zero-time blood samples have been received biweekly. Samples of blood were obtained from the jugular vein using single-use, sterile syringes after disinfecting the region where the blood was obtained. Two sets of samples were created; the first group was stored in 5 ml tubes with the anticoagulant ethyl diamine tetra acetic acid (EDTA) to estimate the white blood cell count and differential white blood cells (WBCs). The remaining blood sample was kept in sterile (10 ml) vacuum tubes. The blood sample was centrifuged for fifteen minutes at 3,000 rpm to extract the serum and measure immunological parameters and globulin content.

### White blood cell count

There are five different kinds of white blood cells were distinguished and measured: neutrophils, monocytes, eosinophils, lymphocytes, and basophils). Furthermore, one drop of blood was applied to the slide and spread out, and the sample was allowed to dry for one minute before being fixed with methyl alcohol to determine the proportion of white blood cells.

The samples were then stained for three to four minutes using (Giemsa stain). After that, three drops of distilled water were added, and they were left on for two minutes. Finally, the samples were washed with tap water and dried. Following that, these were inspected using a light microscope with an oily emersion lens to identify 100 cells, and their percentages were computed as an illustration of [30].

### Enzyme-linked immunosorbent assay (ELISA) test

We used a commercial ELISA kit (IDEXX^®;^) to assess the IgG level, and we simultaneously examined all sera (sera group 1 and 2) with the FMDV ELISA kit.

### Statistical analysis

Using SAS, data statistical analysis was carried out (System for Statistical Analysis—9.1 version). One-way analysis of variance and the least significant differences (LSDs) test were used to assess the significance of mean differences. The indicator of statistical significance is shown by (*p* > 0.05) [31].

## RESULTS

### Description of nanoparticles

**Figure 1.**
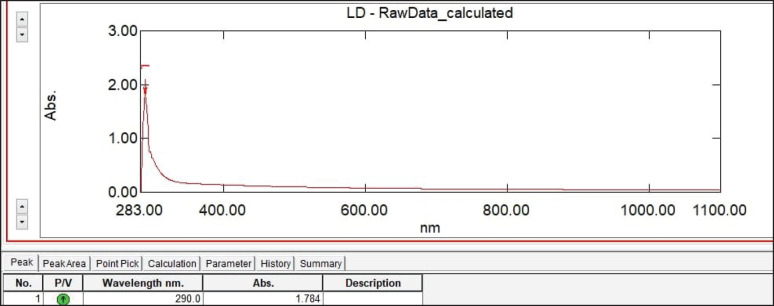
Absorption spectra of selenium nanoparticles isolated from *Pseudomonas.*

With a 190–800 NM Ultra Violet—vis spectrophotometer, the absorbance properties of the isolated selenium nanoparticles were obtained (Fig. 1). The highest absorption, in particular, was observed at about 290 nm.

### *Pseudomonas aeruginosa* bacteria

Selenium nano-particles crystallite structure produced by the biological process was analyzed using the XRD spectrum technique. A common technique for estimating the size and dimensional parameters of crystals is the use of X-rays. The Scherrer equation, which is employed for this purpose, is as follows:

G.S= 0.9λ/β cosθ

G.S is the grain size,

Β is the full width at a half maximum

θ is the diffraction angle

λ is the wavelength for the X-ray source used (1.5406 Å).

In FTIR, the four solutions tested: *P. aeruginosa *culture, supernatant solution after centrifugation of the nutrient cultured with *P. aeruginosa *selenium salt solution (NaHSeO_3_), and the solution of selenium nanoparticles was examined using the chromatographic images obtained during the FTIR test. The results are displayed in (Fig. 3) the examination of IR absorption vibrations based on wave number (cm) allowed for the identification of many zones.

In body weight during the experiment’s 60 days, the group A body weight significantly increased as shown in Table 2.

### Assessment of antibody reactions

Total IgG antibodies in serum samples were quantified using the ELISA enzyme-linked immunosorbent assay. 14, 30, 45, and 60 days following vaccination, serum samples were taken. On day 14, the antibody level was at its maximum. Following vaccination, we observed a statistically significant increase in IgG response in all immunized groups G1 when selenium nanoparticles were given orally at a concentration of 0.1 mg per kilogram of total body weight in comparison to lambs who had only received the FMD vaccine as shown in Figure 4 for three serotypes compare with control group G2.

**Figure 2.**
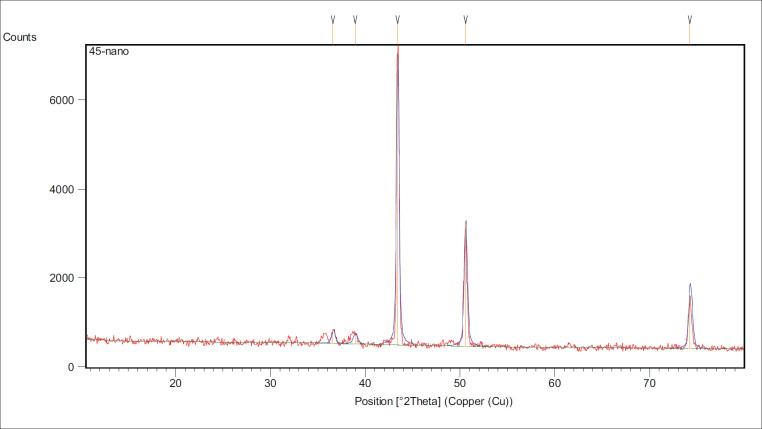
Displays XRD patterns for the Se nanoparticles produced using *P. aeruginosa*.

In the present work, we suggest a possible strategy for using selenium nanoparticles to boost lambs’ immunity. These results can be a consequence of selenium nanoparticles’ greater absorption. Our findings imply that feeding lambs a diet enriched with selenium nanoparticles as shown in Table 3.

## Discussion

The synthesis and surface plasmon vibration of selenium nanoparticles caused the UV spectra to focus in the range of two-three hundred nm [32,33]; moreover, the resonance of surface plasmon of the selenium nanoparticles made by *P. aeruginosa *is represented by the peak with the best definition at 290 nm however the present finding is consistent with findings from studies using other types of bacteria and fungi [34,35]. Additionally, further evidence that the artificial particles were polydisperse came from the peak’s broadening [36].

**Figure 3.**
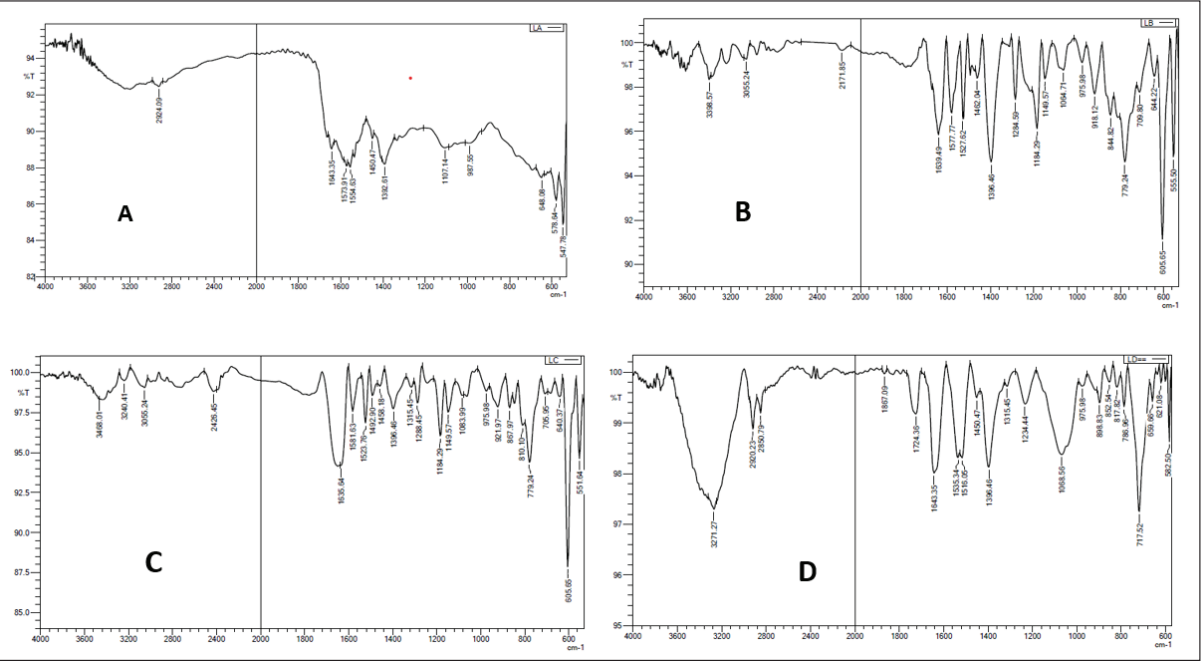
Illustrate the examination of the chromatographic images obtained during the FTIR test for the four solutions that were put to the test: (A) *P. aeruginosa *culture, (B) supernatant solution after centrifugation the nutrient cultured with *P. aeruginosa, *(C) selenium salt solution (NaHSeO3), and (D) selenium nanoparticle solution. Depending on the wave number (cm), different zones were detected in the vibrations induced by different infrared (IR) absorption.

**Table 2.** The effect of Se-nanoparticles on body weight during 60 days.

Groups	Selenium nanoparticles G1	Control G2	LSD
Zero time	E18.97 ± 2.14^a^	B18.71 ± 0.88^a^	5.57
15 days after FMD vaccination	DE21.18 ± 1.82^a^	B20.02 ± 1.09^a^	
30 days after FMD vaccination	CDE23.82 ± 1.78^a^	B20.94 ± 0.94^a^	
45 days after FMD vaccination	CD26.30 ± 1.58^a^	AB23.26 ± 0.32^a^	
60 days after FMD vaccination	AB32.35 ± 1.70^a^	AB26.44 ± 0.67^b^	

Means of different small letters in the same raw are significantly different (*p* < 0.05).. Means of different capital letters in the same column are significantly different (*p* < 0.05).

The findings of this study show that selenium is the primary component of the reduction product in XRD [37] as well as the signal of the elements P, O, and C may be obtained through the extraction of a reducing agent [38].

In the infrared spectra of nanoparticles, the main absorbance bands were at 3271.27, 2920.23, 2850.79, 1867.09, 1724.36, 1643.35, 1535.34, 1516.05, 1450.47, 315.45, 1234.44, 1068.56, 975.98, 898.83, 852.54, 817.82, 786.96, 717.52, 659.66, and 621.08. The vibrations of O–H groups stretched could be responsible for the wide band observed at 3271.27/cm in addition, the vibrations that stretch asymmetrically of amide are what make up the band at 1535.34 and 1516.05 cm/1. However, the 1068.56 cm-1 peaks provide evidence that C–O exists. The sharp band may be caused by carbonyl stretching vibrations in aldehydes, ketones, and carboxylic acids at 717.52 cm^−1^ [35,39]. Many bacterial species probably employ a range of reduction techniques, including selenite reductase enzymes, membrane-associated reductase, periplasmic dissimilatory nitrite reductase, and extracellular reductases (nitrate reductase) [40,41]. These findings showed that these bioactive compounds’ functional groups may potentially operate when synthesizing selenium nanoparticles, as reduction and stabilization agents.

**Figure 4.**
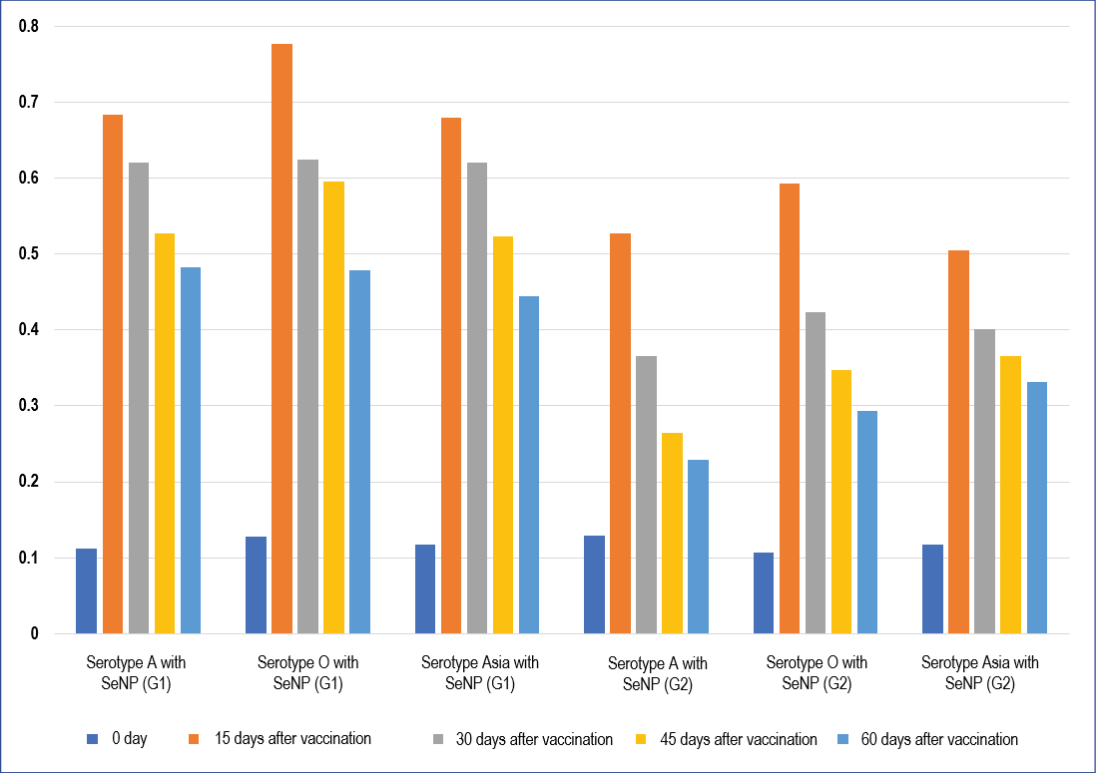
ELISA test for three serotypes of FMD vaccine with administration of selenium nanoparticles in group1 compare with the control group.

The analytical technique of XRD is primarily used to identify crystalline nano materials and can provide details on the size of the nano material and the type of crystal in the compound. Angle Theta-2 value is used in analytical XRD for varied peaks [42].

The prepared selenium nanoparticles demonstrated increased body weight mass gain. The process that selenium might cause a rise in excess weight may be linked to changes in hepatic fatty acid metabolism and energy production by raising the Glut2 transcription in addition to the lipid metabolism-related enzymes [43].

Selenium nanoparticles have been shown to interact with globulin proteins through direct binding interactions, modulation of protein structure and function, enhancement of antioxidant activity, and regulation of gene expression. These interactions suggest a correlation between selenium nanoparticles and globulin, highlighting the potential impact of selenium nanoparticles on immune function and overall health [44,45].

The impact of selenium supplementation or depletion on cellular and humoral immunological reply to various vaccinations has been well studied in studies employing agricultural animals [46]. It is thought that Se’s incorporation into selenium-proteins is responsible for its immunomodulatory actions. Among selenium-proteins, selenoenzymes such as thioredoxin reductases and glutathione peroxidases control the proportion of redox signals to RO species by eliminating too many potentially harmful radicals generated when oxidative damage occurs in immune cells [47,48].

The host’s immunological response against FMD infection depends significantly on humoral immunity. Sandwich ELISA examination of blood samples revealed that, in comparison to a control group, oral administration of selenium nanoparticles followed by injection of the FMD vaccination has significantly improved antibody responses *(p *< 0.05*)*; however, the increased immunological responses found in this study are consistent with earlier studies [49].

**Table 3.** Correlation coefficient between different deferential WBCs after vaccination with the FMD vaccine.

Parameters	Correlation coefficient-r		
	ELISA type A	ELISA type O	ELISA type Asia 1
Lymphocyte	0.783**	0.8602**	0.8674**
Neutrophil	0.7871**	0.9116**	0.8278**
Monocyte	0.35**	0.34**	0.50**
Basophil	0.10 NS	0.11 NS	0.11 NS
Eosinophil	0.02 NS	−0.15 NS	−0.02 NS
Globulin	0.8993**	0.8813**	0.872**

** (*p*<0.01), NS: Non-Significant.

## Conclusion

In this paper, we provide a possible strategy to enhance lamb immunity through the use of selenium nanoparticles. The increased selenium nanoparticle absorption may be the cause of these findings. Our findings indicate that providing lambs with a meal supplemented with selenium nano could potentially stimulate their immune system. Selenium nanoparticles, on the other hand, may facilitate nutritional digestion; accessible selenium, on the other hand, could raise protein creation via enhancing stimulation of many cellular metabolic processes, leading to improved mRNA expression for protein synthesis. As well as food intake and weight gain were both boosted by taking supplements of selenium nanoparticles.

## List of Abbreviations

cm: centimeter; EDTA: ethyl diamine tetra acetic acid; ELISA: enzyme linked immunosorbent assay; FMD: foot and mouth disease; FTIR: Fourier transform infrared spectroscopy; G1: group one; G2: group two; IgG: immunoglobulin type G; IR: Infrared; LSD: least significant differences; NM: nanometer; NMD: nutritional muscular dystrophy; RO: reactive oxygen; Se: selenium; UV: ultra violet; WBCs: white blood cells; XRD: X-ray diffraction analysis.
